# Optimizing Regional Anesthesia for Cancer Patients: A Comprehensive Review of Current Practices and Future Directions

**DOI:** 10.7759/cureus.69315

**Published:** 2024-09-13

**Authors:** Shyamolima Bhuyan, Deepjit Bhuyan, Shubham Rahane

**Affiliations:** 1 Anaesthesiology, Jawaharlal Nehru Medical College, Datta Meghe Institute of Higher Education and Research, Wardha, IND; 2 Anesthesiology, Jawaharlal Nehru Medical College, Datta Meghe Institute of Higher Education and Research, Wardha, IND

**Keywords:** cancer surgery, immune function, oncological outcomes, opioid reduction, pain management, regional anesthesia

## Abstract

Regional anesthesia has emerged as a pivotal component in the perioperative management of cancer patients, offering several advantages over traditional general anesthesia. By providing targeted pain relief and minimizing systemic exposure to opioids, regional anesthesia reduces the risk of opioid-related side effects and enhances postoperative recovery. Regional anesthesia may positively influence oncological outcomes by attenuating the surgical stress response and preserving immune function, potentially reducing cancer recurrence and metastasis. This review comprehensively explores the current practices and benefits of regional anesthesia in the oncology setting, including various techniques such as nerve blocks, epidural anesthesia, and spinal anesthesia. It examines the challenges associated with its application in cancer patients, including technical difficulties and patient-related factors. It evaluates the existing evidence regarding its impact on cancer progression and patient survival. Additionally, the review discusses future directions in the field, emphasizing the need for personalized anesthesia strategies, further research into the long-term effects of regional anesthesia on cancer outcomes, and the development of innovative approaches to enhance its efficacy and safety. By addressing these areas, this review aims to provide a thorough understanding of how to optimize regional anesthesia for cancer patients, ultimately contributing to improved perioperative care and better long-term outcomes.

## Introduction and background

Regional anesthesia, a technique that involves the targeted delivery of anesthetic agents to specific nerves or regions of the body, has become an increasingly important tool in managing cancer patients [1]. Unlike general anesthesia, which affects the entire body, regional anesthesia provides localized pain relief and muscle relaxation, which can be particularly beneficial in oncology. By blocking nerve impulses from a specific area, regional anesthesia minimizes pain. It reduces the need for systemic analgesics, such as opioids, which can have significant side effects, especially in vulnerable cancer patients [2].

The importance of regional anesthesia in cancer care extends beyond pain management. In recent years, there has been growing interest in the potential immunomodulatory effects of regional anesthesia and its impact on cancer recurrence and metastasis [3]. Regional anesthesia may contribute to better oncological outcomes by reducing the body's overall stress response and preserving immune function. Additionally, this approach can decrease the risk of postoperative complications, such as respiratory depression and nausea, thereby facilitating quicker recovery and shorter hospital stays. This is particularly crucial for cancer patients who often require multiple interventions and have compromised health due to their disease and its treatments [4].

This review aims to provide a comprehensive overview of the current practices in regional anesthesia for cancer patients, examining the various techniques used and their respective benefits and limitations. It also explores the evidence linking regional anesthesia to improved cancer outcomes and discusses the potential mechanisms behind these effects. Furthermore, the review identifies challenges in applying regional anesthesia in oncology and proposes future research and clinical practice directions to optimize its use. By synthesizing current knowledge and highlighting areas for further investigation, this review seeks to enhance the understanding and application of regional anesthesia in cancer care, ultimately contributing to better patient outcomes.

## Review

Regional anesthesia techniques for cancer patients

Regional anesthesia plays a crucial role in pain management for cancer patients, particularly during surgical procedures. By targeting specific nerves or areas of the body, these techniques can provide substantial pain relief while minimizing the need for systemic opioids, which are associated with adverse effects. This overview examines various regional anesthesia techniques, including nerve blocks, epidural anesthesia, spinal anesthesia, and continuous regional anesthesia, highlighting their applications, mechanisms, benefits, and limitations [5]. Nerve blocks are localized techniques involving injecting anesthetic agents near specific nerves to provide targeted pain relief. Common nerve blocks used in cancer surgeries include the brachial plexus, paravertebral, and sciatic nerve blocks.

The brachial plexus block is particularly effective for surgeries involving the upper limbs, such as those related to shoulder and upper arm cancers [6]. The paravertebral block is advantageous for thoracic and abdominal surgeries, as it targets the nerves exiting the spinal column, providing pain relief for chest and upper abdomen procedures. Similarly, the sciatic nerve block is employed for lower limb surgeries and is especially beneficial for patients with pelvic tumors or cancers of the lower extremities [7]. The primary indications for nerve blocks in cancer surgeries are centered around localized pain management. These blocks are especially useful when systemic analgesics are insufficient or when patients experience significant pain due to tumors affecting specific anatomical regions. For example, intercostal nerve blocks may be used for chest wall pain, while celiac plexus blocks can relieve pain associated with abdominal cancers, such as pancreatic cancer. By delivering targeted analgesia, nerve blocks can enhance patient comfort and improve surgical outcomes [8].

Epidural anesthesia is a widely used technique that involves injecting anesthetic agents into the epidural space surrounding the spinal cord. This method is particularly effective in major cancer surgeries, including thoracic and abdominal procedures, where it can provide profound pain relief. The mechanism of action involves blocking nerve signals in the targeted region, leading to decreased pain perception and improved patient comfort during and after surgery. One of the key advantages of epidural anesthesia over general anesthesia is its ability to reduce opioid requirements [5]. By minimizing the need for systemic opioids, epidural anesthesia lowers the risk of opioid-related side effects, such as respiratory depression and constipation. Furthermore, this technique can enhance postoperative recovery by alleviating pain, facilitating earlier mobilization, and promoting a quicker return to normal activities. Regional anesthesia techniques, including epidural anesthesia, may have immune benefits by reducing the stress response associated with surgery, thereby preserving immune function in cancer patients [9].

Spinal anesthesia is another effective regional anesthesia technique suited for lower abdominal and pelvic procedures. This method involves injecting anesthetic agents into the cerebrospinal fluid, resulting in rapid onset and profound analgesia. Spinal anesthesia is often used in surgeries such as prostatectomies, hysterectomies, and other procedures involving the lower abdomen [10]. When comparing spinal anesthesia to other regional techniques, it is important to consider the specific surgical site and patient factors. While spinal anesthesia provides a quick onset and dense analgesia, its use is generally limited to surgeries below the umbilicus. In contrast, nerve blocks, such as the paravertebral block, can offer broader coverage for thoracic and abdominal surgeries. The choice between these techniques often depends on the type of surgery, the patient's medical history, and the desired level of anesthesia [11].

Continuous regional anesthesia is an advanced technique that involves the placement of catheters for prolonged analgesia. This method includes continuous nerve blocks and epidural infusions, allowing for sustained pain control following surgery. By providing consistent analgesia over an extended period, continuous regional anesthesia can significantly enhance patient comfort and improve recovery experiences [12]. The benefits of continuous regional anesthesia are substantial, particularly in postoperative pain management. By reducing the need for systemic opioids, it decreases the risk of opioid-related side effects, such as nausea, vomiting, and constipation, which are common in cancer patients. However, there are limitations to this approach. The risk of infection at catheter sites is a concern, as is the potential for catheter-related complications, including block failure or inadequate analgesia if the catheter dislodges [13]. The indications, advantages, and limitations of regional anesthesia techniques for cancer patients are summarized in Table 1.

**Table 1:** Regional anesthesia techniques for cancer patients: indications, advantages, and limitations

Technique	Description	Indications in cancer surgery	Advantages	Limitations
Nerve blocks [14]	The injection of local anesthetics near specific nerves to block pain in a targeted area.	Used in surgeries like breast cancer, limb surgeries, and head and neck cancers.	Provides targeted pain relief, reduces opioid consumption, and facilitates early mobilization.	Potential for incomplete block, nerve damage, and requires expertise.
Epidural anesthesia [15]	The injection of anesthetic agents into the epidural space surrounding the spinal cord.	Common in thoracic, abdominal, and pelvic cancer surgeries.	Excellent pain control reduces stress response and allows for continuous infusion.	Risk of hypotension, infection, and technical difficulty in patients with prior surgeries or radiation.
Spinal anesthesia [16]	The injection of an anesthetic into the subarachnoid space produces a dense block below the injection site.	Primarily used for lower abdominal, pelvic, and lower limb cancer surgeries.	Rapid onset, effective analgesia, and minimal drug use.	Shorter duration compared to epidural, risk of hypotension, and post-dural puncture headache.
Continuous regional anesthesia [17]	It uses catheters to continuously deliver local anesthetics to nerve bundles or epidural space.	Applicable in surgeries requiring prolonged analgesia, such as extensive abdominal or thoracic procedures.	Sustained pain relief, adjustable dosing, and reduced opioid requirement.	Close monitoring is required, and catheter displacement and infection are risks.

Benefits of regional anesthesia in cancer patients

One of the primary advantages of regional anesthesia for cancer patients is its ability to provide effective pain relief while minimizing the need for opioids. Opioids, although widely used for managing postoperative pain, can have significant side effects, including respiratory depression, nausea, vomiting, and constipation [18]. Regional anesthesia techniques, such as epidural and paravertebral blocks, offer effective pain management without requiring high doses of opioids. This reduction in opioid use can lead to fewer opioid-related side effects and potentially improve postoperative recovery. Additionally, better postoperative pain control with regional anesthesia may contribute to faster recovery times and shorter hospital stays for cancer patients [12].

Another potential advantage of regional anesthesia in cancer patients is its possible impact on the immune system and the body's response to surgical stress. Animal studies have suggested that regional anesthesia may help protect cell-mediated immunity and reduce the surgical stress response, potentially affecting cancer recurrence and metastasis [19]. The proposed mechanisms include reducing the neuroendocrine stress response and preserving natural killer cell function. However, evidence from randomized controlled trials and meta-analyses shows no significant benefit of regional anesthesia on recurrence-free survival or overall survival compared to general anesthesia in cancer patients. The existing evidence is limited, and further research is needed to better understand the potential immunomodulatory effects of regional anesthesia in the context of cancer surgery [20].

Regional anesthesia may also positively impact surgical outcomes for cancer patients. Regional anesthesia, particularly thoracic epidural analgesia, can mitigate the stress response to surgery and promote gastrointestinal recovery in gastric cancer patients [21]. This is particularly important because gastrointestinal complications can significantly contribute to morbidity following cancer surgery. Additionally, multimodal analgesic strategies, including regional anesthesia, have been associated with improved postoperative outcomes, such as reduced complications and enhanced recovery. By incorporating regional anesthesia into a multimodal approach, healthcare providers may optimize pain management, decrease opioid consumption, and potentially enhance overall surgical outcomes for cancer patients [22]. The key benefits of regional anesthesia for cancer patients are summarized in Table 2.

**Table 2:** Key benefits of regional anesthesia in cancer patients

Benefit	Description	Implications
Effective pain management [23]	Provides targeted pain relief by blocking specific nerves, reducing the need for systemic analgesics such as opioids.	Enhances patient comfort and decreases opioid-related side effects such as nausea, vomiting, and sedation.
Reduced opioid consumption [24]	Minimizes the use of opioids, which are commonly associated with significant side effects and potential for dependency.	Lowers the risk of opioid-induced complications, including respiratory depression and postoperative ileus.
Immunomodulatory effects [25]	It may help preserve immune function by reducing the surgical stress response and inflammation associated with systemic anesthesia.	Potentially decreases cancer recurrence and metastasis, leading to improved long-term survival.
Decreased postoperative complications [26]	Lowers the risk of complications such as respiratory issues, cardiovascular events, and infection due to better physiological stability and reduced systemic drug use.	Contributes to faster recovery, reduced intensive care unit (ICU) stays, and shorter overall hospital stays.
Improved surgical outcomes [27]	Enhances surgical conditions by providing adequate muscle relaxation and reducing blood loss, especially in major surgeries.	Facilitates safer surgical procedures and potentially better overall outcomes for cancer surgeries.
Enhanced recovery and rehabilitation [28]	Allows quicker mobilization and rehabilitation post-surgery by reducing pain and enabling early participation in physical therapy.	This leads to a shorter recovery period, reduced hospital costs, and a quicker return to daily activities.

Challenges and considerations in regional anesthesia for cancer patients

Regional anesthesia offers substantial benefits for pain management in cancer patients, but it also presents unique challenges that must be carefully navigated to ensure optimal outcomes. These challenges can be categorized into patient-related factors, tumor-related considerations, and technical difficulties, each significantly influencing the anesthetic management strategy [29]. One of the primary challenges in administering regional anesthesia to cancer patients is the presence of comorbidities, coagulopathy, and altered anatomy. Many cancer patients have underlying health conditions, such as cardiovascular disease, diabetes, or respiratory disorders, which can complicate anesthesia management [30]. For example, patients with cardiovascular issues may be at an increased risk of complications during surgery, requiring vigilant monitoring and potentially restricting the use of certain anesthetic techniques. Coagulopathy, which can result from the cancer itself or anticoagulant therapy, raises concerns about the risk of bleeding during and after regional block placement [31]. This necessitates a comprehensive preoperative evaluation and may limit the use of techniques like epidural anesthesia. Additionally, anatomical changes caused by tumor growth or prior surgical interventions can make placing regional blocks more difficult, requiring anesthesiologists to modify their techniques and approaches [32].

Psychological factors also significantly influence the choice and effectiveness of regional anesthesia. Cancer patients often experience heightened anxiety and fear related to their diagnosis and treatment, which can affect their preferences regarding anesthesia. Some patients may prefer regional techniques to minimize opioid use and avoid associated side effects, while others may be uncomfortable with the idea of remaining conscious during the procedure. Understanding these psychological factors and addressing patient preferences is essential for fostering a positive patient experience and effective pain management [33]. The location and type of tumor are critical factors that influence the choice of anesthesia. Regional anesthesia techniques must be customized to the specific surgical approach and the anatomical considerations associated with the tumor. For instance, tumors located in the thoracic region may benefit from thoracic epidural anesthesia, which provides effective analgesia while minimizing systemic opioid use. In contrast, lower abdominal tumors may be better managed with paravertebral blocks. The anesthesiologist must carefully evaluate the tumor’s characteristics and the planned surgical approach to determine the most appropriate anesthetic technique [34].

Another significant consideration is the potential risk of tumor seeding or spreading during the administration of regional anesthesia. There is ongoing debate in the medical community regarding the risk of cancer cell dissemination during block placement. Tissue manipulation during block placement could theoretically lead to the spread of malignant cells, raising concerns about the safety of regional anesthesia in certain situations. However, the clinical significance of this risk remains unclear, and further research is needed to establish safe practices and guidelines for regional anesthesia in cancer patients [35]. Technical challenges also present substantial obstacles to successfully implementing regional anesthesia in cancer patients. Previous surgeries or radiation therapy can result in anatomical changes, scarring, and altered tissue planes, complicating the placement of regional blocks.

Anesthesiologists must be adept at assessing these changes and may need to employ advanced techniques or alternative approaches to achieve effective anesthesia. This requires expertise, experience, and a thorough understanding of the patient’s surgical history and current anatomical considerations [30]. Advanced imaging techniques, such as ultrasound guidance, have become invaluable in overcoming some of these technical challenges. Ultrasound can enhance the accuracy of block placement, improve safety, and reduce the risk of complications by providing real-time visualization of anatomical structures. However, the availability of ultrasound technology and specialized training requirements can pose challenges in some clinical settings. Ensuring that anesthesiologists are proficient in these advanced techniques is crucial for optimizing the use of regional anesthesia in cancer patients [36]. The challenges and considerations in implementing regional anesthesia for cancer patients are summarized in Table 3.

**Table 3:** Challenges and considerations in implementing regional anesthesia for cancer patients

Category	Challenges	Considerations
Patient-related factors [37]	Comorbidities (e.g., cardiovascular disease, diabetes)	Pre-anesthetic assessment to identify risks and optimize patient condition.
	Coagulopathy and bleeding risks	Careful evaluation of coagulation status and potential need for blood products.
	Altered anatomy due to previous surgeries or radiation therapy	Use advanced imaging techniques (e.g., ultrasound guidance) for accurate needle placement.
	Psychological factors and patient preferences	Thorough preoperative counseling to address anxieties and set expectations about anesthesia options.
Tumor-related considerations [4]	Tumor location affecting regional anesthesia approach (e.g., spinal vs. epidural vs. nerve block)	Customized anesthesia plans based on tumor type, location, and extent.
	Potential for tumor seeding or spread due to invasive procedures	Minimize needle insertion through tumor-affected areas and consider non-invasive approaches when possible.
Technical challenges [38]	Difficulty in performing regional blocks due to anatomical changes (e.g., scar tissue, edema)	Training in advanced regional anesthesia techniques and equipment (e.g., nerve stimulators, continuous catheters).
	Need for precision in needle placement to avoid complications	Utilization of real-time imaging techniques like ultrasound to improve safety and accuracy.
	Equipment availability and technological limitations in certain settings	Ensuring access to and maintenance of necessary anesthesia equipment and technology, particularly in resource-limited settings.
Perioperative management [39]	Balancing analgesia with cancer patients’ need for clear mental status during the postoperative period	Selecting appropriate dosages and monitoring protocols to maintain patient comfort without excessive sedation.
	Interaction of regional anesthesia with concurrent chemotherapy or radiation therapy	Coordination with the oncology team to optimize the timing of anesthesia relative to other cancer treatments to minimize adverse interactions and optimize patient safety.

Current evidence on regional anesthesia and cancer outcomes

The potential benefits of regional anesthesia in improving cancer outcomes have been a subject of ongoing research and debate. Regional anesthesia, particularly when combined with general anesthesia, may be associated with lower cancer recurrence rates. For example, a meta-analysis indicated that patients who received regional anesthesia in addition to general anesthesia had a reduced risk of cancer recurrence, with an odds ratio of 0.71, suggesting a possible protective effect of regional anesthesia against cancer recurrence, especially in prostate cancer patients [20]. This proposed benefit is thought to be due to the systemic action of local anesthetics, which might exert direct antitumor effects. Local anesthetics could inhibit cancer cell proliferation, reduce metastasis, and induce apoptosis, contributing to a lower recurrence rate [20]. However, the overall evidence remains conflicting and inconclusive.

A comprehensive review found no significant difference in recurrence-free survival between patients receiving regional anesthesia and those undergoing general anesthesia alone. Furthermore, a meta-analysis concluded that the adjunctive use of regional anesthesia did not significantly reduce cancer recurrence rates in surgical oncology. The discrepancies in study outcomes are likely due to the heterogeneity of study designs, varying methodologies, small sample sizes, and differences in cancer types and anesthetic techniques used, contributing to the ongoing debate about the efficacy of regional anesthesia in improving long-term outcomes in cancer patients [20]. The theoretical basis for the potential benefits of regional anesthesia in cancer outcomes includes its ability to preserve immune function and reduce the surgical stress response. The surgical stress response can activate the hypothalamic-pituitary-adrenal axis, further suppressing the immune system, which may facilitate cancer cell survival and proliferation. Regional anesthesia has been proposed to attenuate this stress response by reducing the release of catecholamines and stress hormones, thereby preserving immune function.

Local anesthetics used in regional anesthesia may modulate innate and adaptive immune responses, potentially impacting tumor cell survival and recurrence [40]. Clinical evidence regarding the influence of regional anesthesia on immune function and cancer outcomes is still evolving. Regional anesthesia may help maintain immune integrity during the perioperative period, potentially reducing the risk of cancer recurrence by preserving natural killer (NK) cell function and limiting the immunosuppressive effects of surgery and anesthesia. However, the evidence remains inconclusive and often contradictory. While some observational studies and smaller trials have reported benefits, larger randomized controlled trials (RCTs) have not consistently demonstrated a significant impact of regional anesthesia on cancer recurrence or overall survival. The current literature emphasizes the need for further investigation, particularly large-scale RCTs, to better understand how different anesthetic techniques affect immune function and subsequent cancer recurrence. Such studies would help clarify these relationships and inform clinical practice more effectively, potentially leading to optimized anesthetic strategies for cancer patients [41].

Regional anesthesia in specific cancer surgeries

Regional anesthesia is increasingly recognized as a critical component in the perioperative management of cancer patients, with its application varying across different types of cancer surgeries, each presenting unique considerations and benefits. Below, we explore the specific uses of regional anesthesia in breast, thoracic and abdominal, head and neck, and pelvic and urogenital cancer surgeries. In breast cancer surgery, regional anesthesia techniques such as paravertebral blocks and pectoral nerve blocks (PECS I and II) have gained popularity for their effectiveness in managing postoperative pain. These techniques are particularly beneficial for procedures like mastectomy and lumpectomy, where effective pain control is crucial for patient comfort and recovery. Paravertebral blocks target the nerves supplying the breast, providing analgesia while minimizing the need for systemic opioids, which can cause adverse effects [4].

Research indicates that regional anesthesia can significantly reduce the incidence of chronic pain syndromes, which affect a considerable proportion of patients post-surgery. Estimates suggest that between 20% to 50% of individuals may experience chronic pain following a mastectomy. Although some laboratory evidence suggests that regional anesthesia could potentially lower cancer recurrence rates by mitigating the stress response associated with surgery, large-scale randomized trials have not consistently demonstrated a significant difference in recurrence rates between patients receiving regional versus general anesthesia. This underscores the need for further investigation into the long-term effects of these techniques on cancer outcomes [42].

In thoracic and abdominal cancer surgeries, regional anesthesia plays a crucial role in enhancing postoperative recovery. Techniques such as epidural blocks and paravertebral blocks are commonly used to provide effective pain relief. These methods not only alleviate pain but also reduce the reliance on opioids, which can lead to complications such as respiratory depression and prolonged recovery times [43]. The benefits of these regional techniques extend beyond pain management and contribute to improved postoperative outcomes. Patients receiving regional anesthesia often experience lower rates of postoperative nausea and vomiting (PONV), significantly enhancing their comfort and satisfaction. Additionally, by attenuating the neurohumoral stress response associated with surgical trauma, regional anesthesia may preserve immune function, potentially benefiting long-term recovery. Overall, integrating these techniques into thoracic and abdominal cancer surgeries has shown promising results in facilitating quicker recoveries and shorter hospital stays [44].

In head and neck cancer surgeries, regional anesthesia techniques such as cervical plexus blocks are employed to provide targeted pain control and minimize the need for systemic opioids. This approach is particularly advantageous in this anatomical region, where effective pain management is critical due to the complexity of the surgical procedures and the potential for significant postoperative pain. By reducing opioid consumption, regional anesthesia can help mitigate the side effects commonly associated with opioid use, such as nausea, sedation, and constipation. However, the use of regional anesthesia in head and neck surgeries is not without its challenges. The intricate anatomy of the neck can pose risks for complications, such as nerve injury or hematoma formation, and variability in individual patient anatomy can affect the efficacy of the blocks. Additionally, achieving adequate analgesia can be difficult due to the complex nerve pathways involved.

Despite these challenges, ongoing advancements in imaging techniques and regional anesthesia protocols continue to improve the safety and effectiveness of these interventions [45]. For pelvic and urogenital cancer surgeries, spinal and epidural anesthesia are frequently used to enhance pain management and facilitate recovery. These regional techniques provide substantial analgesia during and after surgery, reducing postoperative pain and enabling quicker mobilization. The ability to reduce opioid consumption is particularly important in this patient population, as opioids can cause a range of side effects that may complicate recovery [46]. Spinal and epidural anesthesia has been shown to improve functional outcomes, allowing patients to regain their mobility more rapidly and potentially reducing the length of hospital stays. By providing effective pain control, these techniques enhance patient comfort and contribute to a more positive overall surgical experience. As research continues to explore the impact of regional anesthesia on postoperative outcomes in pelvic and urogenital surgeries, evidence increasingly supports its role in optimizing recovery for cancer patients [47].

Future directions in regional anesthesia for cancer patients

Regional anesthesia for cancer patients is poised for substantial advancements in the coming years. A major area of innovation is the ongoing development of nerve localization and imaging guidance techniques. The integration of ultrasound technology has already transformed regional anesthesia by enabling more accurate nerve localization and block placement. Advanced techniques, such as ultrasound-guided central neuraxial and peripheral nerve blocks, hybrid transducers, and real-time 3D imaging, further enhance the efficacy and safety of these procedures. Additionally, research is focused on developing new local anesthetics and adjuvants that can extend the duration of analgesia and improve patient outcomes. Innovations like liposomal formulations and advanced drug delivery systems are being explored to relieve pain with fewer side effects [48].

Another important future direction in regional anesthesia for cancer patients is the shift toward personalized medicine. This approach will increasingly emphasize customizing anesthesia techniques based on individual patient profiles and tumor characteristics, aiming to optimize pain management by considering each patient's unique physiological and pathological aspects. There is also potential for utilizing genetic and molecular markers to predict individual responses to anesthesia. This could lead to the development of more effective and safer anesthesia plans tailored to the biological characteristics of specific tumors and patient genetics [49].

Despite these promising advancements, there is a pressing need for large-scale, RCTs to determine the long-term effects of regional anesthesia on cancer outcomes, including recurrence rates and overall survival. Future research should focus on understanding how regional anesthesia affects not only immediate postoperative recovery but also long-term cancer outcomes, such as its potential influence on immune function and tumor recurrence [4]. The future of pain management in cancer patients will likely involve a multimodal approach that integrates regional anesthesia with systemic analgesics and non-pharmacological methods. By combining various pain management strategies, healthcare providers can enhance the overall recovery experience for cancer patients, addressing both the physical and psychological aspects of pain and recovery. This holistic approach aims to improve pain relief while minimizing the use of opioids and their associated side effects [50]. Future directions for enhancing regional anesthesia in cancer care are summarized in Table 4.

**Table 4:** Future directions for enhancing regional anesthesia in cancer care

Future direction	Description	Potential impact
Innovations in regional anesthesia techniques [51]	Advances in nerve localization and imaging guidance (e.g., ultrasound, nerve stimulators) and the development of new local anesthetics and adjuvants.	Improved precision and effectiveness of regional anesthesia techniques, reducing complications and enhancing patient outcomes.
Personalized medicine approaches [49]	Tailoring anesthesia techniques based on individual patient profiles, tumor characteristics, and genetic markers.	Enhanced patient-specific care, optimizing anesthesia effectiveness and minimizing side effects.
Research on cancer outcomes [20]	Large-scale, randomized controlled trials to investigate the impact of regional anesthesia on cancer recurrence, metastasis, and survival rates.	Better understanding of the role of regional anesthesia in long-term cancer outcomes and potential reduction in recurrence rates.
Multimodal approaches [52]	Combining regional anesthesia with other analgesic modalities (e.g., systemic analgesics and non-pharmacological approaches) to manage pain and reduce opioid use.	More comprehensive pain management strategies, minimizing opioid-related side effects, and improving recovery.
Technological advancements [53]	Development of advanced regional anesthesia devices and techniques, such as automated delivery systems and integration with telemonitoring technologies.	Increased safety, consistency, and patient monitoring capabilities, leading to better patient management.
Exploration of new indications [54]	Expanding the use of regional anesthesia beyond traditional cancer surgeries to include other procedures and palliative care settings.	Broader application of regional anesthesia, improving quality of life for more patients across different stages of cancer care.

## Conclusions

Regional anesthesia offers significant benefits in the care of cancer patients, providing effective pain management while minimizing the adverse effects associated with systemic analgesics like opioids. Its potential to improve surgical outcomes by reducing postoperative complications and potentially influencing cancer recurrence rates has made it a valuable tool in oncological surgery. Despite these advantages, the application of regional anesthesia in cancer care faces challenges, including patient-related factors, technical difficulties, and varying levels of evidence supporting its impact on long-term cancer outcomes. As the field continues to evolve, further research is needed to better understand the mechanisms by which regional anesthesia may affect cancer progression and to refine techniques to maximize patient safety and efficacy. Future studies should focus on large-scale, RCTs to clarify the role of regional anesthesia in different cancer types and surgeries. By advancing knowledge and practice in this area, healthcare providers can optimize anesthetic strategies to improve cancer patients' immediate and long-term outcomes.
